# Diversity and Composition of the Gut Microbiota in the Developmental Stages of the Dung Beetle *Copris incertus* Say (Coleoptera, Scarabaeidae)

**DOI:** 10.3389/fmicb.2020.01698

**Published:** 2020-07-24

**Authors:** Pablo Suárez-Moo, Magdalena Cruz-Rosales, Enrique Ibarra-Laclette, Damaris Desgarennes, Carmen Huerta, Araceli Lamelas

**Affiliations:** ^1^Red de Estudios Moleculares Avanzados, Instituto de Ecología A.C., Xalapa, Mexico; ^2^Red de Ecoetología, Instituto de Ecología A.C., Xalapa, Mexico; ^3^Red de Biodiversidad y Sistemática, Instituto de Ecología A.C., Xalapa, Mexico

**Keywords:** dung beetles, gut microbiota, developmental stages, PICRUSt2 analysis, KOs

## Abstract

Dung beetles are holometabolous insects that feed on herbivorous mammal dung and provide services to the ecosystem including nutrient cycling and soil fertilization. It has been suggested that organisms developing on incomplete diets such as dungs require the association with microorganisms for the synthesis and utilization of nutrients. We describe the diversity and composition of the gut-microbiota during the life cycle of the dung beetle *Copris incertus* using 16S rRNA gene sequencing. We found that *C. incertus* gut contained a broad diversity of bacterial groups (1,699 OTUs and 302 genera). The taxonomic composition varied during the beetle life cycle, with the predominance of some bacterial genera in a specific developmental stage (Mothers: *Enterobacter* and *Serratia*; Eggs: *Nocardioides* and *Hydrogenophaga*; Larval and pupal stages: *Dysgonomonas* and *Parabacteroides*; offspring: *Ochrobactrum*). The beta diversity evidenced similarities among developmental stages, clustering (i) the adult stages (mother, male and female offsprings), (ii) intermediate developmental (larvae and pupa), and (iii) initial stage (egg). Microbiota differences could be attributed to dietary specialization or/and morpho-physiological factors involved in the transition from a developmental stage to the next. The predicted functional profile (PICRUSt2 analysis) for the development bacterial core of the level 3 categories, indicated grouping by developmental stage. Only 36 categories were significant in the SIMPER analysis, including the metabolic categories of amino acids and antibiotic synthesis, which were enriched in the larval and pupal stages; both categories are involved in the metamorphosis process. At the gene level, we found significant differences only in the KOs encoding functions related to nitrogen fixation, uric acid metabolism, and plant cell wall degradation for all developmental stages. Nitrogen fixation and plant cell wall degradation were enriched in the intermediate stages and uric acid metabolism was enriched in mothers. The data reported here suggested the influence of the maternal microbiota in the composition and diversity of the gut microbiota of the offspring.

## Introduction

Insects have symbiotic associations with bacterial communities that affect their development, ecology, and evolution (Feldhaar, 2011; Engel and Moran, 2013; Schwab et al., 2016; Shapira, 2016). The associated microorganisms constitute the microbiota and can serve as a reservoir of additional genes and functions for insect survival (Feldhaar, 2011). For instance, beneficial symbionts facilitate the digestion and production of limited nutrients (Dillon and Dillon, 2004; Douglas, 2009) and protect to the insect-host from pathogens (Engel and Moran, 2013). The composition of the insect gut microbiota has received great attention, it has been demonstrated that the developmental stage (Chen et al., 2016), diet type (Colman et al., 2012; Franzini et al., 2016), host taxonomy (Colman et al., 2012; Kolasa et al., 2019), environment (Ng et al., 2018), and social interactions (Martinson et al., 2012) are important factors affecting the composition and diversity of the microbial communities in the insect gut.

Despite the increased interest in the study of insect gut microbiota (Engel and Moran, 2013), the high species diversity from dung beetles (Ridsdill-Smith and Simmons, 2009) and the ecological functions provided by them (Nichols et al., 2008), the bacterial communities associated to their gut microbiota broadly remained largely unresolved (Thiyonila et al., 2018). Previous studies have reported the role of mutualistic symbionts present in the beetles intestine, facilitating the digestion of dung, mainly by cellulose degradation (Halffter and Matthews, 1971). Recent studies reported the vertical transmission of specific bacteria from the female to its offspring via maternal secretions and a specialized structure, termed pedestal, in the brood ball (Estes et al., 2013). The pedestal is made by maternal excrement and serves as an anchor to eggs into brood ball, and as food source upon hatching of the larva (Byrne et al., 2013; Estes et al., 2013). The importance of the pedestal microbiota in the developmental characteristics (adult body size, time to adulthood, larval mass) and the environmental contingency, was reported in the dung beetle *Onthophagus gazella* (Schwab et al., 2016). Parker et al. (2019) evaluated the role of pedestal microbiota in the host development. They found that the swapping of maternal pedestal between *O. gazella* and *O. sagittarius*, reduces the growth, affects survival, and delays the developmental time.

There is limited knowledge in the dung beetle’s bacterial microbiota, in particular on its diversity and composition during the insect life cycle. Differences in the gut bacterial communities associated with life stages have been reported for several insect species like bark beetles (Briones-Roblero et al., 2017) flies (Wang et al., 2018), beetles (Arias-Cordero et al., 2012), and lepidopteran (Chen et al., 2016). However, the only one report in dung beetles in the genus *Euoniticellus* determined that the taxonomic abundance profiles in the microbiota in larvae, male and female parents were grouped according to developmental stage (Shukla et al., 2016). The authors detected an enrichment in genes involved in cellulose degradation in the larval gut compared to adults. This is thought to be associated with the dietary specialization of each developmental stage (Shukla et al., 2016).

*Copris incertus* is a holometabolous insect with four developmental stages (egg, larva, pupa, adult) each with a characteristic morphology (Klemperer, 1986; Halffter and Matthews, 1996). This dung beetle is coprophagous, and dung digger (Cruz and Huerta, 1998) with a maternal care that affects offspring survival (Halffter and Matthews, 1996). After dung burial, the female pairs with the male and prepared the nest, where the brood ball is collocated for containing an egg (Halffter and Matthews, 1996). Most of the studies regarding the genus *Copris* have examined the life history (Klemperer, 1986), parental care (Klemperer, 1982; Tyndale-Biscoe, 1984; Halffter and Matthews, 1996), reproductive biology and behavior (Cruz and Huerta, 1998), while the taxonomic and functional diversity in its microbiota is lacking.

The goal of the present study was to describe and compare the gut microbiota of *C. incertus* in different stages of its life cycle: female parent, egg, larvae, pupa, male and female offspring. We hypothesized that the changes in the diversity and composition of the gut microbiota and its metabolic functions are associated with the host development. This is the first study of the microbial diversity in the gut of a dung beetle during its full life cycle.

## Materials and Methods

### Samples Collection and Generation of 16S rRNA Amplicons

*Copris incertus* was reared under laboratory conditions, and adult males and females belonging to third-generation were mated, and provisioned with soil and fresh cow dung and placed in cylindrical plastic terraria [see details in Halffter and Matthews (1996)]. Mothers, eggs, third instar larva, pupa, males, and females offspring were collected from three families. Entire guts (from proventriculus to rectum) were dissected and collected from all developmental stages, except the eggs, which were completely macerated. Samples were surface sterilized with washes of 90% ethanol, PBS, and 0.1% tween 20 for 1 min each. One sample per developmental stage per family was processed (Supplementary Table S1). Genomic DNA was extracted using QiAamp Fast DNA stool Mini Kit following the manufacturer’s instructions.

The V4 region of the 16S rRNA gene was sequenced in a 2×300 bp paired-end run using the Illumina Miseq platform. The 16S rRNA raw sequencing results have been deposited in the Sequence Read Archive (SRA^1^) under accession numbers PRJNA603992^2^.

### Quality Filters and Sequence Analysis

Sequencing reads obtained from the MiSeq run were processed using QIIME2 pipeline (ver. 2018.8.0) (Bolyen et al., 2018). The “tools import” and “demux” plugins were used to create the “artifact file” and demultiplex the paired-end sequencing reads. Sequences were quality-filtered with a Phred score Q20 and sequences at least 400 bp in length using the Deblur algorithm (Amir et al., 2017). High-quality sequences were clustered into operational taxonomic units (OTUs) based on 97% pairwise identity against the Greengenes database (gg_13_8_otus). The “uchime-denovo” and “filter-features” methods were used to identify and excluded the chimeric sequences. OTUs were taxonomically classified using the Greengenes database (ver. 13_8) and the “feature-classifier” plugin with the “classifier-sklearn” method. In order to increase taxonomic resolution, sequences that were not taxonomically classified at the genus level were blasted against the NCBI database and Silva 16S rRNA database (release 132). OTUs assigned to chloroplast, mitochondria, and archaea, and sequences that did not have at least ten counts across all samples were removed. The filtered OTUs (filtered OTUs table) were rarefied based on the number of sequences from the library with the lowest sequencing depth within each comparison set.

### Bacterial Composition and Diversity in the Gut Microbiota

A total of 18 samples of the dung beetle *Copris incertus* (Supplementary Table S1) were used to test the effect of the developmental stage in the composition and diversity of the gut bacterial microbiota. For the taxonomic diversity analysis, the total OTUs (“OT”) (filtered and rarefied OTUs), defined as those OTUs present in at least one sample, and the developmental stage bacterial core (“DC”) (OTUs or genera shared by all the samples of the same developmental stage) were used. For functional diversity, only DC was considered.

QIIME2 was used to estimate and visualize alpha-diversity (observed OTUs, Shannon index, and Faith PD) of total OTUs (“OT”), and to test differences among group comparisons using Kruskal-Wallis test. Differences in the bacterial community among life stages (beta-diversity) were calculated with the Bray-Curtis distance (Bray and Curtis, 1957) using the filtered and rarefied OTU table. Differences between all groups and pairwise communities were calculated with a permutational multivariate analysis of variance (PERMANOVA) in QIIME2 (Bolyen et al., 2018).

Dissimilarities in taxonomic diversity among developmental stages were visualized by NMDS ordination with a Bray-Curtis distance matrix of taxon relative abundances using the VEGAN v1.17–2 Package in R (Oksanen et al., 2018). Bar plots and a heatmaps were built to visualize the taxonomic diversity (at the genus level) found in the total OTUs from the life stages, using the R package gplots v.3.0.1 (Warnes et al., 2016). To visualize DC OTUs and taxa shared among the developmental stages an Upsetplot was constructed using R package UpsetR (Lex et al., 2014). A rarefaction curve was computed directly using QIIME2 (Bolyen et al., 2018).

### Functional Prediction of the *C. incertus* Microbiota

To predict the metabolic functional profiles of the bacterial communities in each developmental stage, PICRUSt2 (the Phylogenetic Investigation of Communities by Reconstruction of Unobserved States) version 2 was used (Douglas et al., 2019). A table containing the predicted gene family-counts per sample based on orthologous groups and identifiers as constructed with Kyoto Encyclopedia of Genes and Genomes (KEGG), using the DC reads abundance. The KEGG identifiers were classified at level 3, and the categories unrelated to bacterial metabolism and physiology were removed. Dissimilarities across the relative frequencies in the filtered categories were illustrated with NMDS using Bray-Curtis distance matrix. The effect of the development stages in the dissimilitude matrix was tested by PERMANOVA, using the VEGAN v1.17–2 Package in R (Oksanen et al., 2018). Similarity percentages analyses (SIMPER) were performed to identify the categories that contribute to dissimilarities among developmental stages (Oksanen et al., 2018). The categories at level 3 with significant differences in the SIMPER analysis were used to generate a heatmap using the function heatmap.2 from the R package gplots v.3.0.1 (Warnes et al., 2016).

To determine differences between the relative abundance of the KOs associated with KEGG pathway maps of four specialized beneficial functions in the insect nutrition (Nitrogen fixation, Uric acid metabolism, Iron uptake, plant cell wall degradation, see Supplementary Table S2), a boxplot using R package ggplot2 was generated (Ginestet, 2011). Differences among developmental stages were tested using Kruskal–Wallis tests. Significant differences between pairs were determined using pairwise Wilcoxon as a *post hoc* test with the correction of Benjamini–Hochberg false discovery rate (Benjamini and Hochberg, 1995). Functional contributions of various taxa to different KOs were computed with command –metagenome_contrib of PICRUSt2 (Douglas et al., 2019) and were visualized by barplots.

## Results

### Bacterial Composition and Diversity of the Gut Microbiota

We analyzed the bacterial microbiota from dung beetle *Copris incertus* in different developmental stages using 16S rRNA amplicons. A total of 855,407 high-quality and filtered sequences and 1,701 OTUs were obtained from the 18 assayed samples, with a median of 48,194 reads and 236 OTUs per sample (Supplementary Table S1). Rarefaction curves of the “observed OTUs” from the filtered OTUs table showed a saturating number of OTUs, indicating adequate sampling for all samples (Supplementary Figure S1). From the normalized OTU data (normalized count = 16,538 reads) 1,699 OTUs were obtained and classified at the main taxonomic rank, 100% were assigned to a kingdom level, 97% to phylum, 95% to class, 90% to order, 74% to family and 51% to genera (Supplementary Figure S2).

**FIGURE 1:**
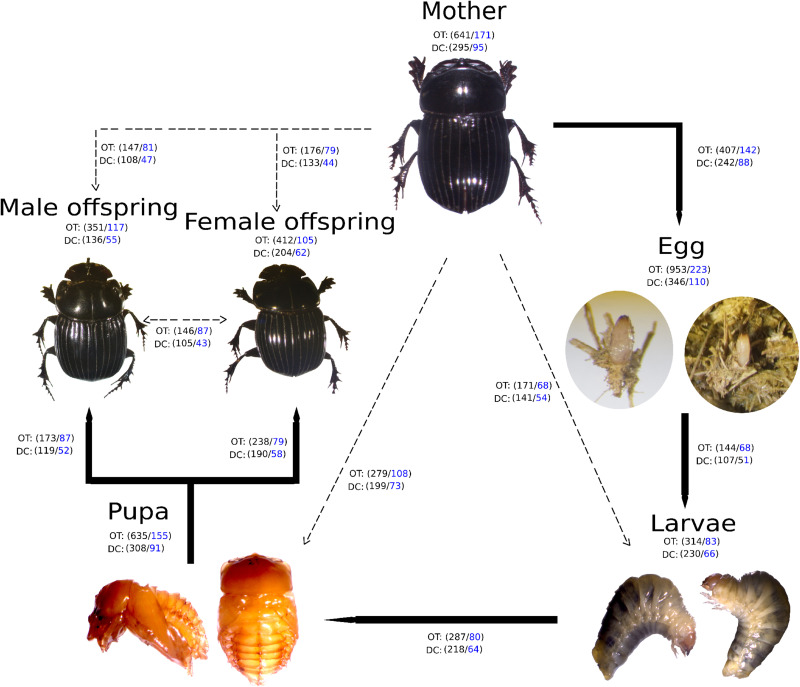
Developmental stages of *Copris incertus*. Total OTUs (“OT”) and the developmental stage bacterial core (“DC”) with the corresponding number of OTUS (in black) and genera (in blue) are indicated for each developmental stage. Lines indicate the OTUs and genera shared among a developmental stage with to the next (solid) and the mother (broken).

**FIGURE 2:**
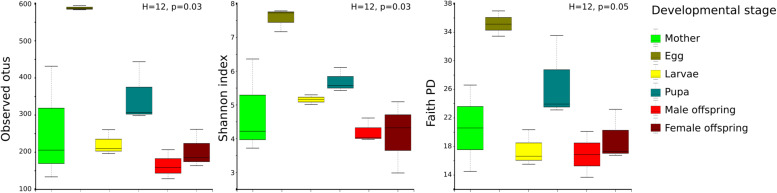
Distribution of alpha diversity of the developmental stages as measured by observed species, Shannon index, and Faith PD. The metrics were based on the total OTUs (OT). Each color represents a developmental stage.

A high bacterial diversity (1,699 OTUs and 302 genera, total OTUs “OT”; 476 OTUs and 128 genera, development’s bacterial core “DC”) was found in the gut of *C. incertus*, which varied according to the developmental stage (Figure 1). The egg stage presented the highest richness in number of OTUs (OT = 953; DC = 346) and genera (OT = 223; DC = 110) (Figure 1), and in the three alpha diversity metrics, with a range of 583–594 to observed OTUs, 7.2–7.7 Shannon index, and 16–20 in Fait PD index. However, only the observed OTUs and Shannon index estimates were significantly different compared to other developmental stages (*p*-value = 0.03 for both) (Figure 2). The analysis of the taxonomic diversity from the DC, revealed that most OTUs (87%) and genera (90%) were shared between some developmental stages, and 29 OTUs and 25 genera were shared by all the samples. Only mothers and eggs had 2 and 60 private OTUs, and 1 and 12 exclusive genera, respectively (Supplementary Figure S3). Larva had the smallest richness as determined by the total OTUs (314 OTUs and 83 genera), and male had the lowest numbers in the DC (136 OTUs and 55 genera) (Figure 1).

**FIGURE 3:**
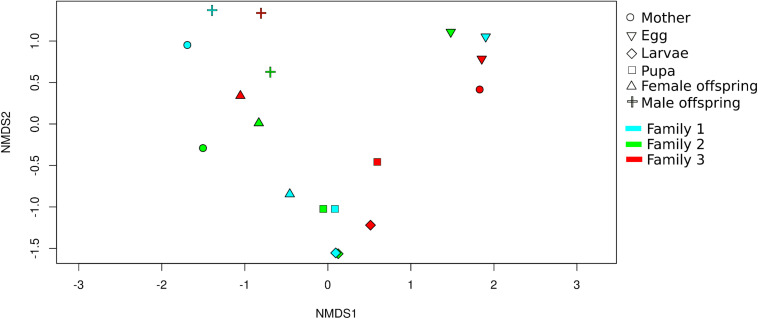
Beta diversity analysis of *C. incertus* developmental stages. Non-metric multidimensional scaling (NMDS) plot based on Bray-Curtis distance of the 1699 OTUs (total OTUs). Each symbol represents the bacterial community in a single dung beetle sample and the color the family of origin.

Non-metric multidimensional scaling analysis (NMDS) showed that developmental stages determined the bacterial community clustering which was corroborated by permutational multivariate analysis of variance (PERMANOVA) (*F* = 4.9, *p* < 0.001) (Figure 3). Also, clustering of adult stage samples (mother, male and female offspring), intermediate developmental samples (larvae and pupa), and initial stage samples (egg) were observed. The sample MOT3 was grouped with the three egg samples. This can be explained by the high number of OTUs shared (350 OTUs/81% of its total OTUs) with the egg samples.

**FIGURE 4:**
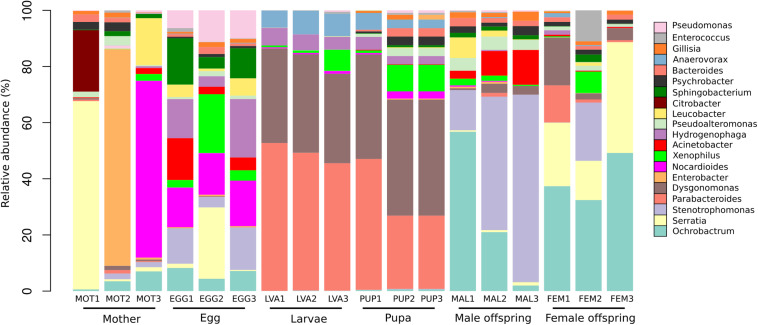
Taxonomic composition of the 20 most abundant bacterial genera in the gut microbiota of the different developmental stages. The percentage of relative abundance of the bacterial genera is represented by different colors.

A total of 19 phyla, 44 classes, 84 orders, 162 families, and 302 genera were detected in the 1,699 OTUs. The phyla Proteobacteria (48%), Bacteroidetes (29%), Firmicutes (14%), Actinobacteria (7%), Verrucomicrobia (0.3%) were the five most abundant. A total of 302 bacterial genera were found among the different developmental stages. The 20 most abundant genera represented 51% of the total reads (Figure 4). At the genus level, the analysis of the bacterial composition showed the predominance of some bacterial groups by developmental stage.

The mothers gut microbiota included 171 genera, with (median/range of the three *C. incertus* samples) *Enterobacter* (0.71%/0–77%), *Serratia* (1.5%/0.71–67%) and *Nocardioides* (0.30%/0.02–63%) as the most abundant genera. For the microbiota of egg, 223 genera were found, with *Nocardioides* (14.8%/14.2–16.2%), *Hydrogenophaga* (13.9%/3.8–21%) and *Stenotrophomonas* (12.6%/3.7–15.1%) as the most abundant genera (Figure 4). Larval and pupal stages were defined by 83 and 155 genera, respectively, 80 of which were shared by both stages. The dominant taxa for three samples of larva and pupa were *Dysgonomonas* (median = 34% and 41%, respectively) and *Parabacteroides* (median = 49 and 26%, respectively) (Figure 4). The female offspring was defined by 62 genera, with *Ochrobactrum* (median = 37.4%/32.4–49.2%) and *Serratia* (median = 22.6%/13.9–39.4%) as the most abundant genera. Male offspring had 55 genera, with *Stenotrophomonas* (median = 47.6/14.3–66.8%) and *Ochrobactrum* (median = 21%/2–56.8%) as the most abundant. The mother and eggs showed the highest microbiota diversity intra samples (Figure 4).

### Functional Predictions of the Development’s Core Bacterial

5,373 KEGG Orthology groups (KOs) were predicted in the developmental stage bacterial core (“DC”) from the different *C. incertus* samples (PICRUSt2 analysis). At level 3 of KEGG 171 categories associated with 23 categories at level 2 were obtained (Supplementary Table S3). The metabolic categories that exhibited higher KOs frequencies included Metabolism of carbohydrates (mean = 19.5 ± 0.40) and Amino acids (mean = 13.8 ± 0.20), while the categories with the lower frequencies included Transport and catabolism (mean = 0.1 ± 0.10), and Drug resistance antineoplastic (mean = 0.2 ± 0.01) (Supplementary Table S3).

**FIGURE 5:**
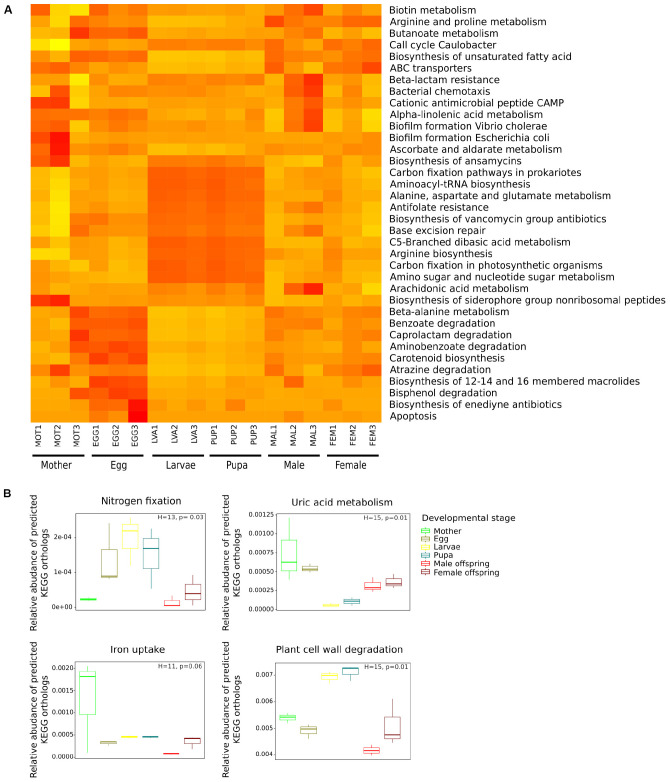
Comparison of predicted KEGG ortholog groups (KOs) count at level 3 **(A)** and gene-level **(B)** by developmental stage. **(A)** Heat map of 36 categories at level 3 significant in the SIMPER analysis, each column corresponds to a *C. incertus* sample, and each row corresponds to a specific category. **(B)** Relative abundance of predicted KEGG orthologs (KOs) involved in nitrogen fixation, uric acid metabolism, iron uptake, plant cell wall degradation.

The NMDS carried out with a Bray-Curtis distance matrix based on the relative frequency of the 171 categories (Supplementary Figure S4), revealed that the functional composition of the samples significantly differed between developmental stages (PERMANOVA, *F* = 3.7, *p* < 0.001). The SIMPER analysis identified 36 categories as the primary drivers of the observed differences between the developmental stages (Figure 5A). Different patterns in the frequencies of these categories by developmental stage were identified. Larva and pupa were more similar (Figure 5A), exhibiting a high frequency of metabolic categories associated to energy metabolism (Carbon fixation by photosynthetic organisms and prokaryotes), translation (Aminoacyl-tRNA biosynthesis), metabolism of carbohydrates (Amino sugar and nucleotide sugar metabolism), biosynthesis of antibiotics (Antifolate resistance, Biosynthesis of ansamycins, and Biosynthesis of vancomycin group) and amino acids (Alanine, aspartate, and glutamate metabolism). Other categories at level 3 as “ABC transporters” and “Biosynthesis of unsaturated fatty acids” were less frequent in these developmental stages. Eggs showed a specific group of categories including biosynthesis of antibiotics (Enediyne) and amino acid metabolism (Beta-Alanine); however, these categories were not exclusive of this life stage. The rest of the categories were evenly distributed among the *C. incertus* samples (Figure 5A). Our results suggested that the relative frequency of KOs determines the dissimilarities between life stages, instead of the presence or absence of specific KOs.

The metabolic capabilities: nitrogen fixation and recycling, iron uptake, and degradation of plant cell wall components (cellulose, hemicellulose, lignin, and pectin) were compared between developmental stages based on the relative abundance of specific KOs predicted by PICRUSt2 (Supplementary Table S3). All the comparisons significantly differed between developmental stages except for iron uptake (Figure 5B). Larva and Pupa had a higher relative abundance in KOs involved in nitrogen fixation and plant cell wall degradation than adults, while adults and eggs showed KOs enriched for uric acid metabolism (nitrogen recycling) (Figure 5B). Wilcoxon *post hoc* test indicated that none of the pairwise comparisons between the developmental were significant for the four functions. Significant differences in the relative abundance of KOs associated with cellulose, hemicellulose, and lignin degradation were found between developmental stages. Larva and pupa showed a higher relative abundance of the KOs associated with cellulose and hemicellulose degradation, while initial stages and adult KOs were enriched for lignin degradation (Supplementary Figure S5).

Analyses of the taxa contributing to the four metabolic functions, by PICRUSt2 predictions determined that the plant cell wall degradation function was most diverse in bacterial genera (*n* = 110) followed by iron uptake (*n* = 56), uric acid metabolism (*n* = 40) and nitrogen fixation (*n* = 13) (Supplementary Figure S6). The main taxa which contributed to nitrogen fixation in larva and pupa stages were (median/range of the three *C. incertus* samples) the genera *Anaerosporomusa* (29%/23–35% and 29%/25–30%, respectively) and *Sporomusa* (19%/12–29% and 13%/12–29%, respectively), while in eggs were *Azoarcus* (36%/13–48%) and *Azovibrio* (17%/3–19%) genera, and in adults was the OTU assigned to the family Halobacteriaceae (27%/0–80% mother, 4%/0–100% male offspring, 10%/1–60% female offspring) (Supplementary Figure S6A). In the uric acid metabolism, the main contributors were the genera *Enterobacter*, *Ochrobactrum*, *Nocardioides*, *Acinetobacter*, and *Serratia* in all life stages. In this metabolic function, the egg showed the highest number of taxa associated with this metabolic function (*n* = 48 genera in total) (Supplementary Figure S6B).

For iron uptake, *Dysgonomonas* (48%/45–50% and 51%/49–53%, respectively) and *Parabacteroides* (36%/35–37% and 33%/16–34%, respectively) were identified as the main taxa in larval and pupal stages, *Rhodococcus* (16%/11–42%) and *Pseudomonas* (13%/11–23%) in eggs, *Sporomusa* (0%/0–65%) and *Serratia* (0%/0–48%) in female offspring, *Dysgonomonas* (19%/6–22%) and *Gillisia* (19%/10–20%) in males, and *Enterobacter* (15%/0–97%) and *Serratia* (6%/0.2–92%) in mothers (Supplementary Figure S6C). Regarding, plant cell wall degradation, *Dysgonomonas* (58%/40–59% and 37%/34–61%, respectively) and *Hydrogenophaga* (25%/17–28% and 11%/6–19%, respectively) were the main taxa assisting larva and pupa the genus *Nocardioides* (8%/7–15%) in egg *Ochrobactrum* (0%/0–64%) and *Dysgonomonas* (0%/0–16%) in females offspring, *Ochrobactrum* (34%/4–69%) in male offspring and *Enterobacter* (0.74%/0–86%) and *Serratia* (0.17%/0.07–70%) in mothers. Eggs had the highest number of taxa associated to this metabolic function (Supplementary Figure S6D).

## Discussion

### The Predominance of Bacterial Groups According to the Developmental Stage

We described and compared for the first time the gut microbiota throughout the life cycle of a dung beetle by 16S rRNA gene amplicons. The principal discovery of the present study was that the composition and diversity, as well as the functional potential of the bacterial communities of *Copris incertus* varied across the developmental stages.

The main difference in the gut microbiota among the developmental stages of *C. incertus* was determined by the relative frequencies of the OTUs (and genera) rather than the presence or absence of specific microbes. Similar results had been observed in other dung beetles like *E. intermedius* and *E. triangulatus*, which showed differences in the relative abundance of bacterial families in larval and adult stages (Shukla et al., 2016). Other cases are illustrated by the Malaria Mosquito *Anopheles gambiae* (Wang et al., 2011), and the butterfly *Heliconius erato* (Hammer et al., 2014), which also exhibited differences in the relative abundance of shared taxa mainly among larva and adults.

We found that as with other holometabolous insects the eggs and mothers are more diverse in the number of OTUs, genera, and/or the alpha diversity metrics (Chen et al., 2016; Shukla et al., 2016). High variation in the bacterial composition and diversity observed in the microbiota of these two developmental stages may be due to the colonization of opportunistic bacteria present in the food and environment. The structural properties of the egg may allow colonization of bacteria from the female secretions deposited in the brood chamber or the dung (Shukla et al., 2016). In the present study, however, the brood ball and untreated dung microbiota were not included. The maternal secretions and pedestal have been suggested as a source of bacteria for the brood ball, due to the significant differences (unweighted UniFrac distances) between the brood ball and untreated dung (Shukla et al., 2016). Therefore, maybe that the brood ball could be the source of the bacterial for the colonization of eggs. Another simple explication is that some bacteria present in the female parent secretions or brood balls remained on the egg surface after the sterilization protocol. However, a future sampling from the environment, including cow dung, the pedestal, and brood ball would be required in order to discern the importance of the maternal microbiota and horizontal transmission, in the diversity and composition of the gut microbiota of *C. incertus*.

The beta-diversity analysis in the gut microbiota from *C. incertus* clustered initial (eggs), intermediate (larva and pupa), and adult stages (mother, offsprings). Similar results were reported for the synanthropic fly, *Chrysomya megacephala* (Wang et al., 2018). These changes in the bacterial communities of the gut microbiota throughout the host life cycle might be due to different factors: a) dietary specialization of adult and larvae. Adults feed on small particles of cow dung while larvae feed on their natal brood ball (Holter and Scholtz, 2007; Scholtz, 2008). The brood ball contains significantly smaller particle sizes and a relatively higher Carbon/Nitrogen ratio than the bulk cow dung (Byrne et al., 2013; Shukla et al., 2016). Differences in the amino-acids concentration in the organic matter of the dung, nest, and brood ball for three species of dung beetles were reported (Rougon et al., 1990). Different types of diet could result in different biochemical conditions in the gut supporting the selection of different bacterial communities. Shukla et al. (2016) reported that larvae and adults have different taxonomic and functional potential (PICRUSt2 analysis) associated with dietary specialization and ontogenetic traits intrinsic to each developmental stage. b) morpho-physiological factors involved in the transition from a developmental stage to the next. During the metamorphosis of holometabolous insects, organs, including the gut, overcome anatomical remodeling (Engel and Moran, 2013; Johnston and Rolff, 2015; Hammer and Moran, 2019). In *C. incertus*, the transition from the third larvae to the pupa showed changes in the gut morphology, similar to what is observed in adult stage (C. Huerta, unpublished data). Compared to larvae, the adult’s gut has larger diameter and length and lacks compartmentalization of the hindgut (Halffter and Matthews, 1966; Halffter and Edmonds, 1982; Shukla et al., 2016).

We suggested that these changes in the gut from *C. incertus* determine the predominance of the bacteria groups during its development. However, we further observed that the diversity and composition of the microbiota in pupa was more similar to larva than to other developmental stages, despite the morphological differences of their gut. This result may support the impact of the larval microbiota in the bacterial communities of the pupa. We hypothesized that if *C. incertus* pupa lack immune response, as has been reported for honeybee’s workers and drone pupa (Gätschenberger et al., 2013). Larva would be responsible to provide the microbiota that assists with defense mechanisms against pathogenic attack. This is relevant considering the beetle’s lifestyle (dung inhabitants, where undigested waste materials, as well as pathogenic microbes from the gastrointestinal tract of mammals, are common) (Thiyonila et al., 2018). Studies in other species of dung beetles have reported that the third instar larva constructs a pupal protective shell with a mix of its defecation material and regurgitated fluids (Edwards, 1986; Rougon et al., 1990), which also could explain the similarities found in the microbiota of these developmental stages.

We observed that the pupa shared a high number of OTUs and genera with the adult stages, however, the differences were found at the abundance level. We suggested that the metamorphosis process *per se* affects the dominance of certain microbial groups. In addition, the appearance of new host tissues that could be colonized by a different group of bacteria represents another factor determining the dominance of specific bacterial groups (Hammer and Moran, 2019). Nevertheless, these microbial changes result beneficial to the adult of *C. incertus*, as has been reported for other insects including *Spodoptera littoralis* (Chen et al., 2016) and *Galleria mellonella* (Johnston and Rolff, 2015), and burying beetle *Nicrophorus vespilloides* (Wang and Rozen, 2017).

*Enterobacter* and *Serratia* were the dominant genera in mothers; *Nocardioides* and *Hydrogenophaga* in eggs; *Dysgonomonas* and *Parabacteroides* in larval and pupal stages; *Ochrobactrum* and *Serratia* in the female offspring; and *Stenotrophomonas* and *Ochrobactrum* in the male offspring. *Enterobacter* was the most abundant genus in the gut microbiota of the *Onthophagus taurus* mothers (Estes et al., 2013) and the gut of *Onthophagus dama* adults (Kumari et al., 2018). The genus *Dysgonomonas* was more abundant in the larval stage of *E. intermedius* and *E. triangulatus* (Shukla et al., 2016) and the gut microbiota of two *Pachysoma* MacLeay desert dung beetle species (Franzini et al., 2016).

The results of PICRUSt2 analysis showed that the gut microbiota in the developmental stages could carry various metabolic functions beneficial to the host, as is expected from symbiotic microorganisms, which provide their insects-host with essential nutrients like nitrogen, vitamins, cofactors, and enzymes involved in the detoxification and food processing (Douglas, 2009). The categories at level 3 diversity (L3), were grouped by developmental stage and showed to be more similar between larvae and pupa stages. This indicates the high number of bacterial diversity and L3 categories shared between these developmental stages. Functional differences associated with developmental stages of holometabolous insects have been reported for dung beetles in the genus *Euoniticellus* (Shukla et al., 2016), including plant cell wall degradation (glycoside hydrolases) and nitrogen fixation, and in several KEGG categories at the second hierarchical level (cell motility, membrane transport) for the lepidopteran *Spodoptera littoralis* (Chen et al., 2016).

We found that L3 categories enriched in larva and pupa were involved in functions related to amino-acid metabolism. A high rate of amino acids degradation has been reported for the larval stage from three dung beetle species (Rougon et al., 1990), mainly six amino acids that include alanine, which metabolism was enriched in larval and pupal stages of *C. incertus*. Additionally, the effect of the lack of amino acid in the development and survival of the larva has been reported in the beetle *Tenebrio molitor* (Davis, 1975).

The categories of antibiotics biosynthesis (antifolate resistance, ansamycins, vancomycin) showed a high relative frequency in pupa and larvae stages. This has been previously reported for lepidopteran *Spodoptera littoralis* larva, which contains symbionts responsible for the production of bacteriocins (mundticin KS) that act against the pathogenic strains in genus *Enterococcus* (Shao et al., 2017). It has been suggested that the production of antimicrobial peptides into the pupal gut could prevent infection of pathogenic microbes during the metamorphosis (Hammer and Moran, 2019). We found some categories at level 3 include KOs related to human diseases, including bacteria that colonize plants asymptomatically. Under certain conditions, these pathogens would identify the insect gut as a secondary host before colonizing their primary host (Tyler and Triplett, 2008; Holden et al., 2009). On the other hand, the insect gut microbiota is a source of antimicrobial compounds against human pathogens (Heise et al., 2019).

Except for iron uptake, we found significant differences in the relative abundance of specific metabolic functions among the life stages (at the gene level). The relative abundance of the KOs involved in nitrogen fixation and plant cell wall degradation was higher in the larval and pupal stages than initial and adult stages. Similar results were reported for the KOs involved in glycoside hydrolase enzymes (including plant cell wall degrading enzymes) and nitrogenase in larvae *versus* male and female adults in the genus *Euoniticellus* (Shukla et al., 2016). Contrary to our results, the authors found non-significant differences in the relative abundances of KEGG Orthologs involved in uric acid metabolism (Shukla et al., 2016). In our case, initial and adult stages exhibited a significantly higher relative abundance than the larva and pupa stages.

Our results suggested high activity of the plant cell wall degradation enzymes and nitrogenases in intermediate stages. This could be explained by the content of the larva diet, relatively poor in nitrogen but rich in cellulose (Shukla et al., 2016). The high relative abundance of KOs involved in nitrogen fixation in intermediate stages supports the idea that the high amino acid concentration is essential for the larva of dung beetle species (Rougon et al., 1990). The prediction of KOs associated to plant cell wall degradation enzymes suggested that the dung beetles depend on microorganisms for the complete utilization of nutrients, assuming the gut as a fermentation chamber with cellulose-digesting bacteria (Halffter and Matthews, 1971). Endonucleases activity has been reported *in vivo* for the larva’s microbiota of African dung beetle *Euoniticellus intermedius* (Mabhegedhe, 2017). It has been suggested that the products of cellulose breakdown- pectin and xylan- are essential nutrients for larva development (Shukla et al., 2016). Furthermore, recycling of uric acid may represent a source of nitrogen for initial and adult stages, while for larva and pupa nitrogenase activity may play the main role in the nitrogen acquisition. It remains to be determined whether KOs associated with iron uptake, are associated to a particular metabolic process, protection from iron toxicities, or if it has antagonistic effects against pathogenic bacteria as has been proposed for the microbiota of other insect species (Sonawane et al., 2018).

Experimental and molecular studies have supported the functional potential of a number of bacterial genera in nitrogen fixation, iron uptake, Uric acid metabolism, and plant cell wall degradation (Schäfer et al., 1996; Indiragandhi et al., 2007; Anand et al., 2010; Engel and Moran, 2013; Inoue et al., 2015). *Serratia*, *Enterobacter*, and *Pseudomonas* are among the top 20 most relevant contributors in plant cell wall degradation. Bacterial strains isolated from the gut of *Bombyx mori* in these genera, showed cellulolytic activity, degrading cellulose, xylan, pectin, and starch (Anand et al., 2010). In our study, the genus *Ochrobactrum* was the major participant in plant cell wall degradation in adults and eggs. Bacterial strains of this genus have hemicellulose-degrading activity (1,3-b-Galactanase, b-D-Galactosidase, 1,4-b-xylanase, b-D-Xylosidase) in termite guts (Schäfer et al., 1996). *Dysgonomonas* seemed to be determinant for plant cell wall degradation in larval and pupal gut of *C. incertus*. Strains isolated in the genus *Dysgonomonas* have shown production of acetic acid, lactic acid, propionic acid, succinic acid as the main end-products of glucose fermentation (Sakamoto, 2014). In addition, there is evidence of the involvement of this genus in plant cell wall degradation as indicated by its capability of hydrolyzing cellulose by beta-glucosidase in termite guts (Zhang et al., 2014). The genus *Serratia* was the most important contributor in iron uptake in adult and egg stages. Isolates of this genus from lepidopteran guts were positive for the synthesis of siderophores *in vitro* (Indiragandhi et al., 2007).

For Uric acid metabolism, the genera *Pseudomonas* and *Serratia* were among the top 20 most important contributors. These were able to recycle uric acid *in vitro*, used as a nitrogen source for eggs, larvae, and adults (male and female) of the beetle *Dendroctonus valens* (Morales-Jiménez et al., 2013). The bacterial nitrogen-fixing activity has been reported in insects like termites, cockroaches, ants, aphids, and beetles [see Bar-Shmuel et al. (2020)]; however, none of the contributing genera have been reported as nitrogen fixers in insect gut; with exception of the order rhizobiales, which are known for their ability to fix nitrogen in plants (Masson-Boivin et al., 2009).

The functional potential and taxa contribution that we identified here by PICRUSt2 analysis requires an experimental appraisal to confirm the importance and functions of gut microbiota in the development of *C. incertus*.

### Influence of Mother Microbiota in the Developmental Stages

Our study evidenced that the bacterial diversity and their metabolic functions are shared between the mothers and their offspring. We suggested that maternal care in *C. incertus* has a significant influence on the diversity and composition of the bacterial microbiota of the initial, intermediate, and offspring stages. The parental care in the burying beetle *Nicrophorus vespilloides* has been suggested as a mechanism of gut microbiota transmission from the mother to the offspring, facilitating the colonization of the larval gut (Wang and Rozen, 2017). In the genus *Copris*, maternal care tasks involve the incorporation of excrement and secretions into the mass of dung used for the brood ball, protection of brood ball against opportunistic insects and fungi, and the constant maintenance of the brood ball until the brood emerges (Klemperer, 1982; Tyndale-Biscoe, 1984; Halffter and Matthews, 1996). For *C. incertus* maternal care has been reported to be a key factor for brood development and survival (Halffter and Matthews, 1996). Moreover, we cannot exclude that this maternal behavior acts as a mechanism of vertical transmission of the bacterial communities to later stages, as well as favoring the offspring survival. *C. incertus* mothers anchored the egg to the brood ball with a pedestal, as other *Copris* species (Bang et al., 2004). This structure made from mother’s feces have not the maternal gift found for dung beetles in the genus *Euoniticellus* (Shukla et al., 2016), and *Onthophagus* (Estes et al., 2013). However, we do not exclude the vertical transmission of the microbiota, and the promoting growth and development of the *C. incertus* offsprings by this maternal structure as have been reported for other dung beetles (Estes et al., 2013; Schwab et al., 2016; Shukla et al., 2016). The influence of the mother in the offspring has been reported in the dung beetle *O. taurus*. In this species, larva and pupa shared several identical OTUs with their mother (Estes et al., 2013). Our results support early studies reporting the importance of maternal microbiota in the development and survival of the dung beetle (Halffter and Matthews, 1971; Byrne et al., 2013; Estes et al., 2013; Schwab et al., 2016; Shukla et al., 2016; Parker et al., 2019).

## Data Availability Statement

The data can be found here: https://www.ncbi.nlm.nih.gov/bioproject/PRJNA603992.

## Author Contributions

AL, CH, MC-R, and EI-L: conception and design of the work. CH and AL: funding acquisition. PS-M and AL: analysis and interpretation of data for the work and drafting the work. DD: revising it critically for important intellectual content. All authors approved the manuscript.

## Conflict of Interest

The authors declare that the research was conducted in the absence of any commercial or financial relationships that could be construed as a potential conflict of interest.

## Footnotes

1 http://www.ncbi.nlm.nih.gov/sra

2 https://www.ncbi.nlm.nih.gov/bioproject/PRJNA603992
