# Supplementary material for: Diversity and Composition of the Gut Microbiota in the Developmental Stages of the Dung Beetle Copris incertus Say (Coleoptera, Scarabaeidae)
Source: Front Microbiol. 2020 Jul 24;11:1698. doi: 10.3389/fmicb.2020.01698 (PMC7393143; doi:10.3389/fmicb.2020.01698)
Supplement: Supplementary file 1 [file Data_Sheet_1.pdf]

Supplementary Material

Supplementary Figures

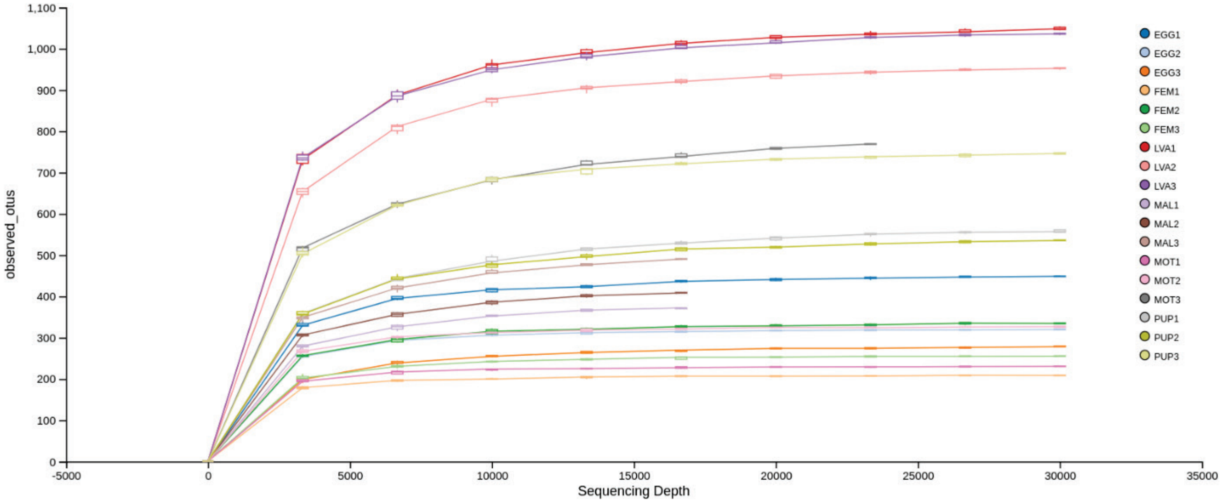

Supplementary Figure 1. Rarefaction curves of the observed OTUs in the 18 dung beetle samples. Each color represents a *C. incertus* sample.

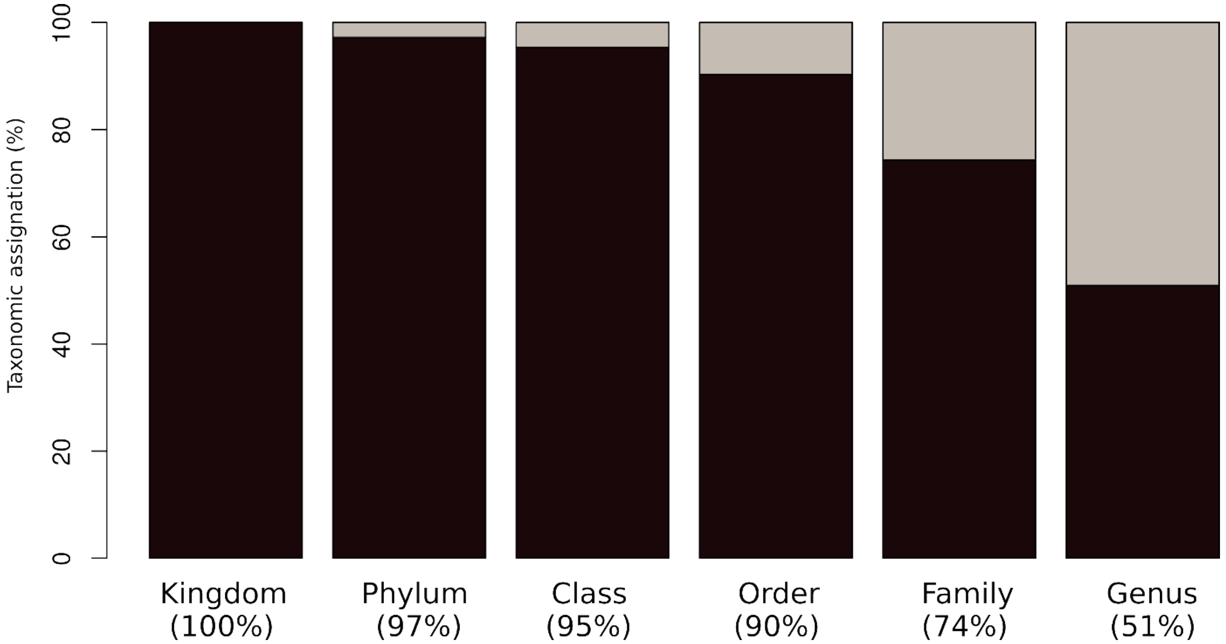

Supplementary Figure 2. Taxonomic assignation for the developmental stages of *C. incertus* OTUs. The percentage bacterial OTUs assigned to a taxonomic level are indicated in parenthesis.

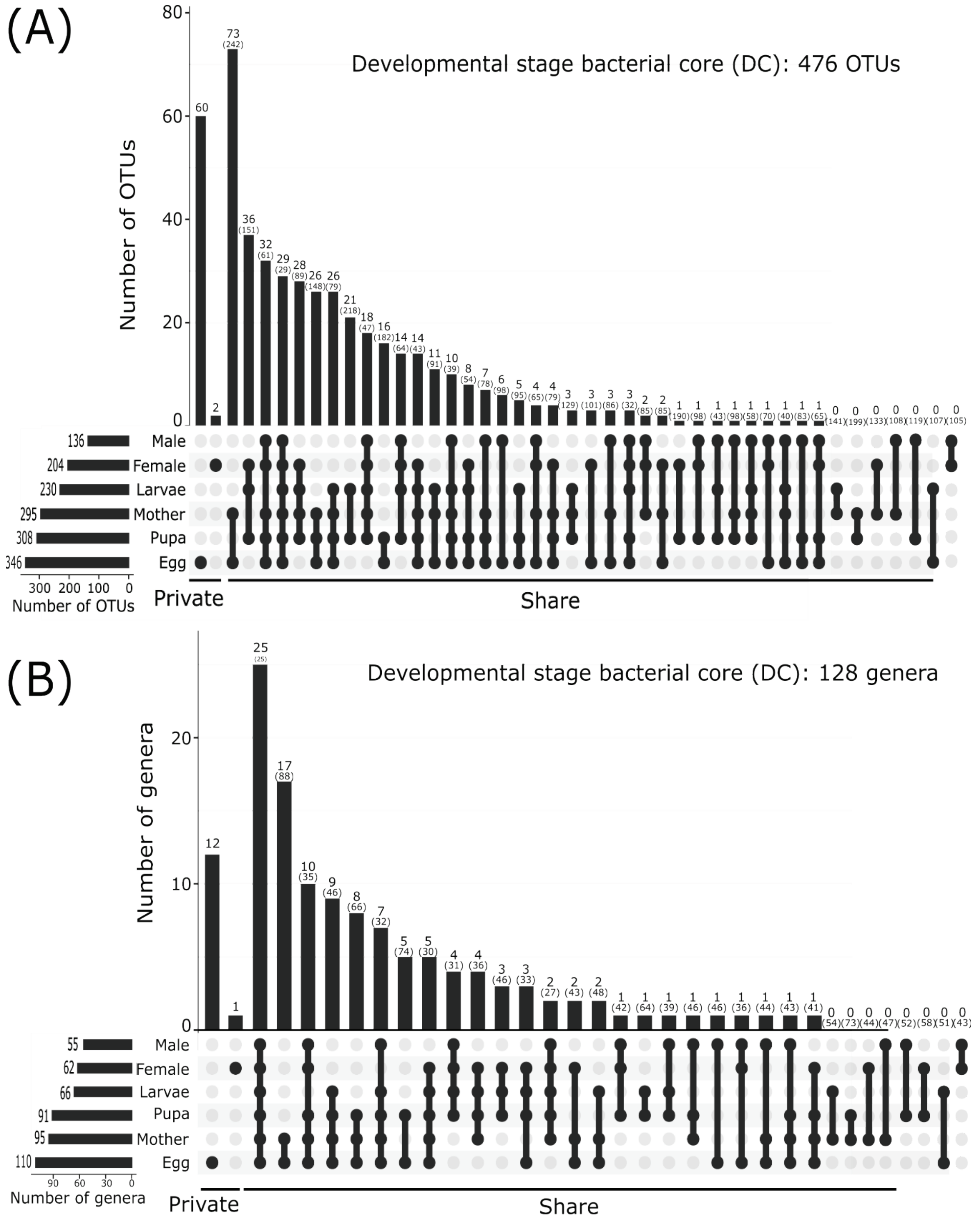

Supplementary Figure 3. Number of OTUs (A) and genera (B) of the developmental stage bacterial core ("DC") shared and private among the different developmental stages. The number of total OTUs and genera for the comparisons is shown in parenthesis at the top of the bars.

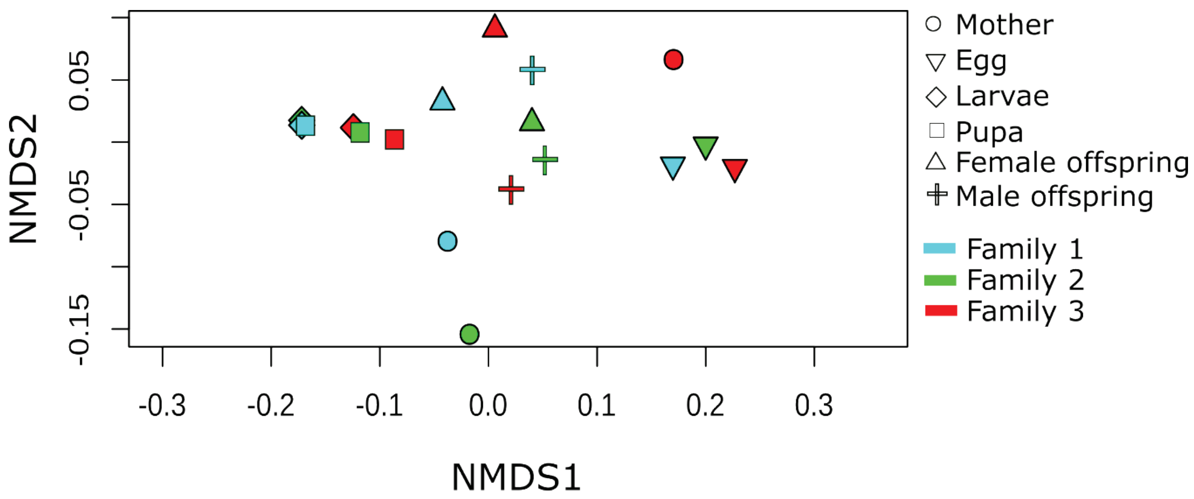

Supplementary Figure 4. Functional clustering of bacterial communities associated with the full life cycle of *C. incertus*. NMDS with Bray-Curtis distance with the 171 KEGG categories (level 3). The developmental stages are represented by different symbols and families by different color.

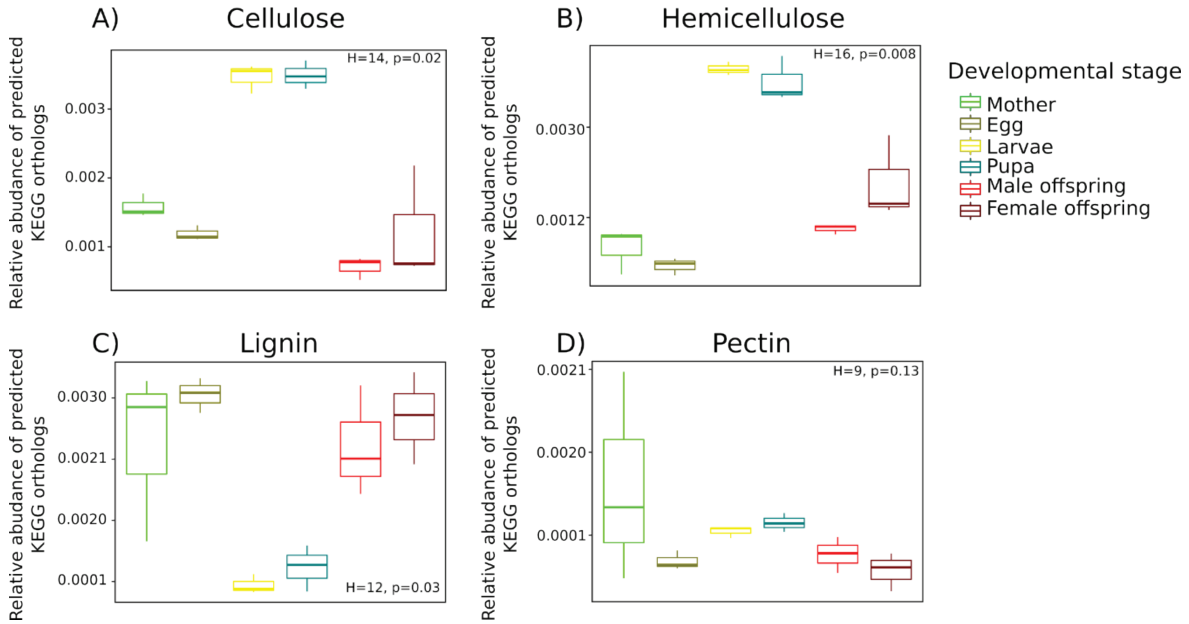

Supplementary Figure 5. Relative abundance of predicted KEGG orthologs (KOs) involved in the biosynthesis pathway of the plant cell wall polysaccharides: Cellulose (A), Hemicellulose (B), Lignin (C), and Pectin (D).

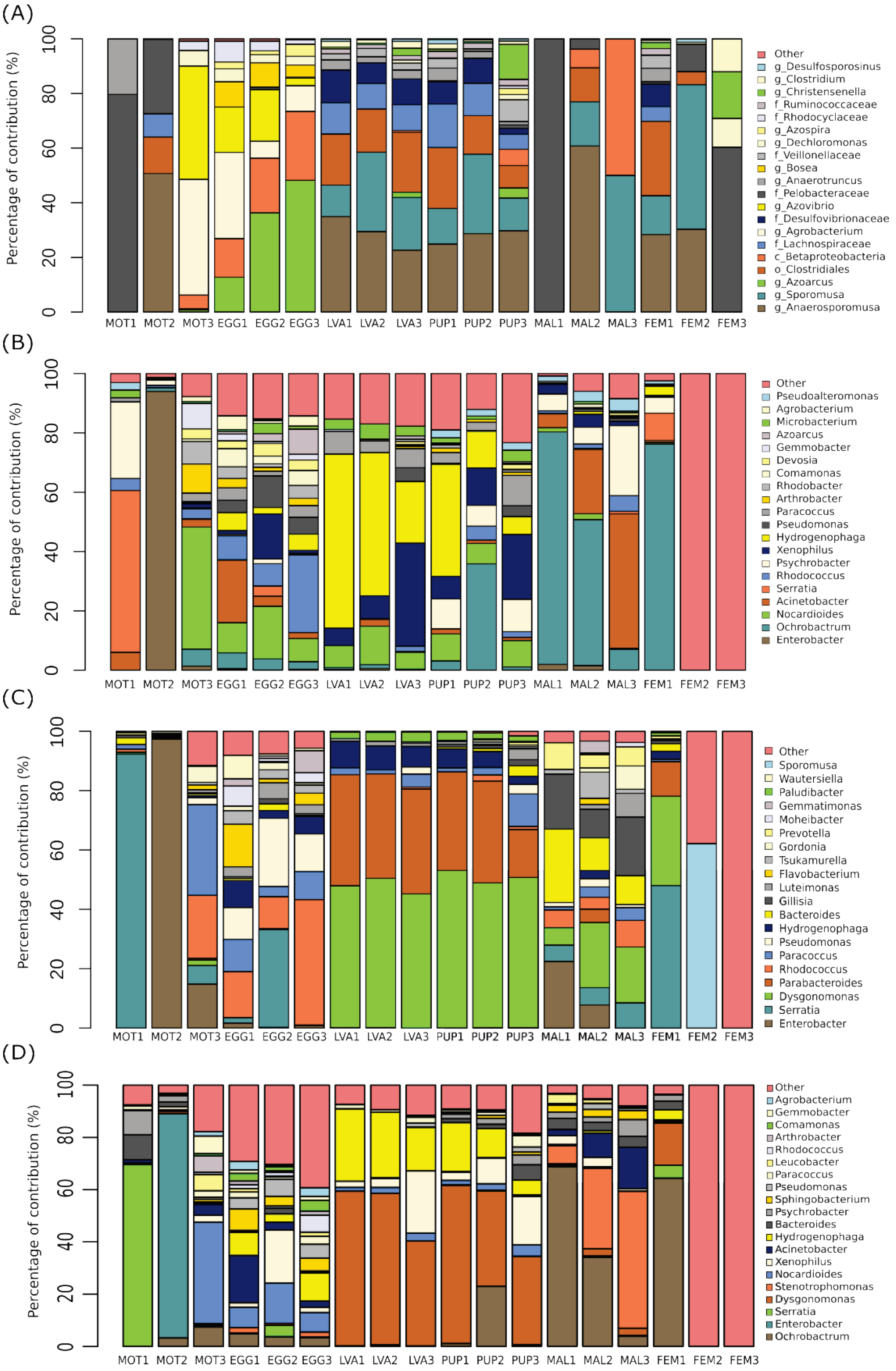

Supplementary Figure 6. Functional contribution of the taxa to different KOs to the four metabolic functions: Nitrogen fixation (A), Uric acid metabolism (B), Iron uptake (C), Plant cell wall degradation (D). The taxa associated with the functions are represented by different colors.

Supplementary Table 1. OTUs obtained from *Copris incertus* developmental stages and families. The number of reads and OTUs obtained for each individual, and the number of OTUs for the sum of different OTUs by developmental stage (OT) and the OTUs shared between all individuals of the same developmental stage (DC) are also shown.

Sample ID	Developmental stage	Family	#Reads	#OTUs	Total OTUs (OT)* #OTUs	Developmental stage bacterial core (DC)* #OTUs
MOT1	Mother	1	62980	134		
MOT2	Mother	2	58931	207	641	295
MOT3	Mother	3	23485	439		
EGG1	Egg	1	47731	620		
EGG2	Egg	2	53644	604	953	346
EGG3	Egg	3	38230	602		
LVA1	Larvae	1	17125	196		
LVA2	Larvae	2	18986	212	314	230
LVA3	Larvae	2	16538	260		
PUP1	Pupa	1	45778	319		
PUP2	Pupa	2	44423	307	635	308
PUP3	Pupa	3	50219	456		
MAL1	Male offspring	1	75739	130		
MAL2	Male offspring	2	70621	212	173	136
MAL3	Male offspring	3	75739	161		
FEM1	Female offspring	1	58968	267		
FEM2	Female offspring	2	48657	185	238	204
FEM3	Female offspring	3	47613	168		
		total	855407	1701	1699	476

* the number of OTUs is based in the normalized OTU table (16538 reads per sample)
